# Supplementary material for: Evaluating algorithmic fairness of machine learning models in predicting underweight, overweight, and adiposity across socioeconomic and caste groups in India: evidence from the longitudinal ageing study in India
Source: PLOS Digit Health. 2025 Nov 26;4(11):e0000951. doi: 10.1371/journal.pdig.0000951 (PMC12654920; doi:10.1371/journal.pdig.0000951)
Supplement: S1 Table — (DOCX) [file pdig.0000951.s001.docx]

**S1 Table. Descriptive Statistics of Key Health Outcomes: Prevalence of Underweight, Overweight, and High Waist Circumference Based on BMI and Waist Measurements**

|  | **Respondent**  **No. (%)** |  |  |  |
| --- | --- | --- | --- | --- |
|  | **Full Sample**  **(N=55,647)** | **Underweight**  **(n=10,315)** | **Overweight/Obesity**  **(n=24,570)** | **High Waist Circumference**  **(n=25,515)** |
| **Age group** |  |  |  |  |
| 45-55 | 20,797(37.37) | 2,802(27.16) | 10,455(42.55) | 10,022(39.28) |
| 55-65 | 17,330(31.14) | 3,096(30.01) | 7,933(32.29) | 8,302(32.54) |
| 65-75 | 12,280(22.07) | 2,790(27.05) | 4,728(19.24) | 5,286(20.72) |
| 75-85 | 4,225(7.59) | 1,247(12.09) | 1,239(5.04) | 1,587(6.22) |
| 85+ | 1,015(1.82) | 380(3.68) | 215(0.88) | 318(1.25) |
| **Gender** |  |  |  |  |
| Male | 25,971(46.67) | 5,002(48.49) | 10,276(41.82) | 6,687(26.21) |
| Female | 29,676(53.33) | 5,313(51.51) | 14,294(58.18) | 18,828(73.79) |
| **Education** |  |  |  |  |
| no schooling | 25,979(46.69) | 6,698(64.93) | 8,622(35.09) | 10,946(42.90) |
| less than 5 years complete | 10,343(18.59) | 661(6.41) | 6,743(27.44) | 5,741(22.50) |
| 5-9 years complete | 12,807(23.01) | 1,660(16.09) | 6,583(26.79) | 6,197(24.29) |
| 10 or more years complete | 6,518(11.71) | 1,296(12.56) | 2,622(10.67) | 2,631(10.31) |
| **Caste** |  |  |  |  |
| Other/no caste | 15,346(27.58) | 1,976(19.16) | 8,492(34.56) | 8,764(34.35) |
| Scheduled caste | 9,817(17.64) | 2,257(21.88) | 3,189(12.98) | 3,175(12.44) |
| Scheduled tribe | 9,393(16.88) | 2,163(20.97) | 3,502(14.25) | 3,875(15.19) |
| Other backward class | 21,091(37.90) | 3,919(37.99) | 9,387(38.21) | 9,701(38.02) |
| **Region** |  |  |  |  |
| North | 9,973(17.92) | 1,397(13.54) | 5,256(21.39) | 5,736(22.48) |
| Central | 7,540(13.55) | 2,249(21.80) | 2,207(8.98) | 2,622(10.28) |
| East | 10,395(18.68) | 2,689(26.07) | 3,421(13.92) | 3,692(14.47) |
| Northeast | 7,428(13.35) | 1,326(12.86) | 2,618(10.66) | 2,418(9.48) |
| West | 7,096(12.75) | 1,147(11.12) | 3,558(14.48) | 3,780(14.81) |
| South | 13,215(23.75) | 1,507(14.61) | 7,510(30.57) | 7,267(28.48) |
| **MPCE** |  |  |  |  |
| Lowest | 11,285(20.28) | 3,341(32.39) | 3,071(12.50) | 3,522(13.80) |
| Lower middle | 11,313(20.33) | 2,535(24.58) | 4,192(17.06) | 4,524(17.73) |
| Middle | 11,124(19.99) | 1,966(19.06) | 4,885(19.88) | 5,142(20.15) |
| Upper middle | 11,025(19.81) | 1,495(14.49) | 5,746(23.39) | 5,812(22.78) |
| Highest | 10,900(19.59) | 978(9.48) | 6,676(27.17) | 6,515(25.53) |
