# Supplementary material for: Evaluating algorithmic fairness of machine learning models in predicting underweight, overweight, and adiposity across socioeconomic and caste groups in India: evidence from the longitudinal ageing study in India
Source: PLOS Digit Health. 2025 Nov 26;4(11):e0000951. doi: 10.1371/journal.pdig.0000951 (PMC12654920; doi:10.1371/journal.pdig.0000951)
Supplement: S2 Table — (DOCX) [file pdig.0000951.s002.docx]

**S2 Table. Definitions and Descriptions of Variables Used in the Analysis**

| **Variable Name** | **Type** | **Definition/Categories** |
| --- | --- | --- |
| **Demographic Variables** |  |  |
| Age | num | Continuous age measured in years. |
| Gender | binary | Categories: Male; Female. |
| Education | category | Categories: No schooling; Less than 5 years complete; 5–9 years complete; 10 or more years complete. |
| Migration status | binary | Categories: Yes (migrated); No (non-migrated). |
| State | category | 35 states in India. |
| Region | category | Categories: North; Central; East; Northeast; West; South. |
| Residence | binary | Categories: Rural; Urban. |
| Religion | category | Categories: Hindu; Muslim; Others. |
| Marital status | binary | Categories: Yes (married); No (unmarried/divorced/widowed). |
| Living alone | binary | Categories: Yes (living alone); No (living with others). |
| **Socioeconomic Variables** |  |  |
| Economic status (MPCE quintile) | category | Categories: Lowest; Lower middle; Middle; Upper middle; Highest. |
| Working status | category | Categories: Currently working; Never worked; Have worked in the past. |
| Type of main job | category | Categories: Agricultural/Fishery/Labor; White-collar job; Others; No current work. |
| Monthly pension | num | Continuous monthly pension amount. |
| Retired officially | binary | Categories: Yes (officially retired); No (not retired). |
| Pension received | binary | Categories: Yes (receiving pension); No (not receiving pension). |
| Caste | category | Categories: General; Scheduled Tribe; Scheduled Caste; Other Backward Class. |
| **Household Characteristics** |  |  |
| Household size | num | Continuous household size. |
| Household headship (female) | binary | Categories: Yes (female head); No (non-female head). |
| Main source of drinking water | category | Categories: Pipe on premises; Other improved sources; Unimproved sources. |
| Time taken to fetch water | binary | Categories: Yes (less than 15 minutes); No (more than 15 minutes). |
| Home ownership | binary | Categories: Yes (own home); No (do not own home). |
| **Health Variables** |  |  |
| **Diagnosed chronic conditions or diseases** |  |  |
| Hypertension | binary | Categories: Yes (diagnosed); No (not diagnosed). |
| Diabetes | binary | Categories: Yes (diagnosed); No (not diagnosed). |
| Cancer | binary | Categories: Yes (diagnosed); No (not diagnosed). |
| Chronic lung disease | binary | Categories: Yes (diagnosed); No (not diagnosed). |
| Chronic heart diseases | binary | Categories: Yes (diagnosed); No (not diagnosed). |
| Stroke | binary | Categories: Yes (diagnosed); No (not diagnosed). |
| Bone/joint diseases | binary | Categories: Yes (diagnosed); No (not diagnosed). |
| Psychiatric problems | binary | Categories: Yes (diagnosed); No (not diagnosed). |
| High cholesterol | binary | Categories: Yes (diagnosed); No (not diagnosed). |
| **Urogenital** |  |  |
| Chronic Renal Failure | binary | Categories: Yes (diagnosed); No (not diagnosed). |
| Incontinence | binary | Categories: Yes (diagnosed); No (not diagnosed). |
| Kidney Stones | binary | Categories: Yes (diagnosed); No (not diagnosed). |
| BPH (Benign Prostatic Hyperplasia) | binary | Categories: Yes (diagnosed); No (not diagnosed). |
| **Immunization** |  |  |
| Influenza vaccine | binary | Categories: Yes (received); No (not received). |
| Pneumococcal vaccine | binary | Categories: Yes (received); No (not received). |
| Hepatitis B vaccine | binary | Categories: Yes (received); No (not received). |
| Typhoid vaccine | binary | Categories: Yes (received); No (not received). |
| Diphtheria and Tetanus (dT) | binary | Categories: Yes (received); No (not received). |
| **Symptom-based health** |  |  |
| Pain | binary | Categories: Yes (reported pain); No (no pain). |
| Sleep problem | binary | Categories: Yes (reported sleep problem); No (no sleep problem). |
| **Vector-borne diseases** |  |  |
| Malaria | binary | Categories: Yes (diagnosed); No (not diagnosed). |
| Dengue | binary | Categories: Yes (diagnosed); No (not diagnosed). |
| Chickungunya | binary | Categories: Yes (diagnosed); No (not diagnosed). |
| **Infectious diseases** |  |  |
| Tuberculosis | binary | Categories: Yes (diagnosed); No (not diagnosed). |
| Urinary tract infection | binary | Categories: Yes (diagnosed); No (not diagnosed). |
| **Others** |  |  |
| Overall Cognition score | binary | Categories: Lowest 10% (Yes); Not lowest 10% (No). |
| Depression (CESD measured) | binary | Categories: Yes (depressed); No (not depressed). |
| Self-rated general health | label | Categories: Very poor; Poor; Fair; Good; Very good. |
| **Functional Limitation Variables** |  |  |
| Vision | binary | Categories: Low vision (Yes); Not low vision (No). |
| Grip strength | num | Continuous measurement of grip strength. |
| Aids used | binary | Categories: Yes (using aids); No (not using aids). |
| ADL | binary | Categories: Yes (difficulty in at least one activity of daily living); No (no difficulty). |
| IADL | binary | Categories: Yes (difficulty in at least one instrumental activity of daily living); No (no difficulty). |
| Use of denture | binary | Categories: Yes (uses dentures); No (does not use dentures). |
| **Behavioral Risk Factors Variables** |  |  |
| Smoking | binary | Categories: Yes (currently smoking); No (not currently smoking). |
| Smokeless | binary | Categories: Yes (using smokeless tobacco); No (not using smokeless tobacco). |
| Alcohol | category | Categories: Lifetime abstainer; Infrequent non-heavy drinker; Frequent/heavy drinker. |
| Physically active | category | Categories: Low; Moderate; High. |
| Yoga/meditation/asana/pranayama | binary | Categories: Yes (engages more than once a week); No (does not engage regularly). |
| Food availability | binary | Categories: Yes (reported severe food constraints); No (no severe food constraints). |
| **Health Service Use and Health Insurance Variables** |  |  |
| Out-patient care | binary | Categories: Yes (any outpatient visit last year); No (none). |
| Inpatient care | binary | Categories: Yes (any inpatient visit last year); No (none). |
| Mean out-of-pocket expenditure on out-patient care in one year prior to the survey | num | Continuous variable representing expenditure. |
| Mean out-of-pocket expenditure on inpatient care in one year prior to the survey | num | Continuous variable representing expenditure. |
| Public Health insurance | binary | Categories: Yes (covered by public insurance); No (not covered). |
| Private Health insurance | binary | Categories: Yes (covered by private insurance); No (not covered). |
| **Family and Social Variables** |  |  |
| Making family decision | binary | Categories: Yes (involved in family decisions); No (not involved). |
| Look after grandchildren | binary | Categories: Yes (responsible for caregiving); No (not responsible). |
| Provide financial support | binary | Categories: Yes (provides financial help); No (does not provide help). |
| Received financial support | binary | Categories: Yes (receives financial help); No (does not receive help). |
| Having family members who are unable to carry out basic daily activities | binary | Categories: Yes (has dependent family members); No (none). |
| Membership in any organization | binary | Categories: Yes (member of organization); No (not a member). |
| Discrimination | binary | Categories: Yes (experienced discrimination); No (no experience of discrimination). |
| Life satisfaction | label | Categories: Low; Medium; High. |
| Receive social benefit | category | Number of benefits received. |
| **Outcome Variables** |  |  |
| BMI category | category | Categories: Underweight (BMI < 18.5); Normal (BMI 18.5–23); Overweight (BMI 23–25); Obesity (BMI > 25). |
| Waist circumference | binary | Categories: Male (> 94 cm); Female (> 80 cm). |
