# Supplementary material for: Evaluating algorithmic fairness of machine learning models in predicting underweight, overweight, and adiposity across socioeconomic and caste groups in India: evidence from the longitudinal ageing study in India
Source: PLOS Digit Health. 2025 Nov 26;4(11):e0000951. doi: 10.1371/journal.pdig.0000951 (PMC12654920; doi:10.1371/journal.pdig.0000951)
Supplement: S3 Table — (DOCX) [file pdig.0000951.s003.docx]

**S3 Table. Comparison of Evaluation Metrics Across Different Machine Learning Models**

1. **Underweight**

|  | **Accuracy**  **(95% CI)** | **Sensitivity**  **(95% CI)** | **Specificity**  **(95% CI)** | **Precision**  **(95% CI)** | **AUROC**  **(95% CI)** |
| --- | --- | --- | --- | --- | --- |
| **Logistic Regression** | | | | | |
| Overall | 0.83 (0.82-0.83) | 0.23 (0.21-0.24) | 0.97 (0.96-0.97) | 0.62 (0.58-0.65) | 0.80 (0.79-0.81) |
| General | 0.88 (0.87-0.89) | 0.13 (0.10-0.17) | 0.99 (0.99-0.99) | 0.66 (0.55-0.76) | 0.80 (0.78-0.83) |
| Scheduled caste | 0.79 (0.78-0.81) | 0.34 (0.30-0.38) | 0.93 (0.92-0.94) | 0.60 (0.54-0.66) | 0.81 (0.78-0.83) |
| Scheduled tribe | 0.79 (0.77-0.81) | 0.25 (0.21-0.29) | 0.96 (0.95-0.97) | 0.67 (0.60-0.74) | 0.79 (0.77-0.81) |
| Other backward class | 0.81 (0.80-0.83) | 0.19 (0.17-0.22) | 0.97 (0.96-0.97) | 0.59 (0.53-0.65) | 0.79 (0.77-0.80) |
| MPCE Lowest | 0.73 (0.72-0.75) | 0.35 (0.31-0.39) | 0.91 (0.89-0.92) | 0.62 (0.57-0.66) | 0.75 (0.73-0.77) |
| MPCE Lower middle | 0.78 (0.77-0.80) | 0.23 (0.19-0.26) | 0.96 (0.95-0.97) | 0.62 (0.55-0.69) | 0.77 (0.75-0.79) |
| MPCE Middle | 0.81 (0.80-0.83) | 0.16 (0.12-0.19) | 0.97 (0.97-0.98) | 0.58 (0.49-0.67) | 0.79 (0.77-0.81) |
| MPCE Upper middle | 0.88 (0.87-0.89) | 0.13 (0.09-0.17) | 0.99 (0.98-0.99) | 0.64 (0.52-0.76) | 0.80 (0.77-0.82) |
| MPCE Highest | 0.92 (0.90-0.93) | 0.10 (0.06-0.15) | 1.00 (0.99-1.00) | 0.77 (0.60-0.92) | 0.80 (0.77-0.84) |
| **Random Forest** | | | | | |
| Overall | 0.82 (0.81-0.82) | 0.09 (0.08-0.10) | 0.99 (0.98-0.99) | 0.61 (0.56-0.67) | 0.78 (0.77-0.79) |
| General | 0.88 (0.87-0.89) | 0.05 (0.03-0.07) | 1.00 (1.00-1.00) | 0.70 (0.53-0.86) | 0.78 (0.76-0.81) |
| Scheduled caste | 0.78 (0.77-0.80) | 0.16 (0.13-0.19) | 0.97 (0.96-0.98) | 0.64 (0.55-0.73) | 0.80 (0.78-0.83) |
| Scheduled tribe | 0.77 (0.75-0.79) | 0.09 (0.06-0.12) | 0.98 (0.98-0.99) | 0.64 (0.52-0.75) | 0.76 (0.73-0.78) |
| Other backward class | 0.80 (0.79-0.82) | 0.07 (0.05-0.09) | 0.99 (0.98-0.99) | 0.55 (0.46-0.64) | 0.76 (0.75-0.78) |
| MPCE Lowest | 0.72 (0.70-0.74) | 0.17 (0.14-0.19) | 0.96 (0.95-0.97) | 0.65 (0.57-0.72) | 0.74 (0.72-0.76) |
| MPCE Lower middle | 0.77 (0.75-0.78) | 0.08 (0.05-0.10) | 0.98 (0.97-0.99) | 0.55 (0.43-0.66) | 0.75 (0.73-0.77) |
| MPCE Middle | 0.81 (0.79-0.82) | 0.05 (0.03-0.07) | 0.99 (0.99-1.00) | 0.58 (0.42-0.74) | 0.77 (0.74-0.79) |
| MPCE Upper middle | 0.88 (0.86-0.89) | 0.05 (0.02-0.07) | 1.00 (0.99-1.00) | 0.63 (0.42-0.84) | 0.76 (0.74-0.79) |
| MPCE Highest | 0.91 (0.90-0.92) | 0.02 (0.00-0.04) | 1.00 (1.00-1.00) | 0.67 (0.20-1.00) | 0.78 (0.75-0.82) |
| **XGBoost** | | | | | |
| Overall | 0.82 (0.81-0.83) | 0.26 (0.24-0.28) | 0.95 (0.95-0.96) | 0.57 (0.54-0.59) | 0.79 (0.78-0.80) |
| General | 0.88 (0.87-0.89) | 0.15 (0.12-0.19) | 0.98 (0.97-0.98) | 0.51 (0.42-0.59) | 0.79 (0.77-0.82) |
| Scheduled caste | 0.80 (0.78-0.82) | 0.39 (0.35-0.44) | 0.92 (0.91-0.94) | 0.61 (0.56-0.67) | 0.80 (0.78-0.82) |
| Scheduled tribe | 0.78 (0.77-0.80) | 0.27 (0.23-0.32) | 0.95 (0.93-0.96) | 0.61 (0.54-0.68) | 0.77 (0.74-0.79) |
| Other backward class | 0.81 (0.79-0.82) | 0.23 (0.20-0.26) | 0.95 (0.94-0.96) | 0.52 (0.47-0.57) | 0.78 (0.76-0.79) |
| MPCE Lowest | 0.73 (0.71-0.75) | 0.37 (0.34-0.41) | 0.88 (0.87-0.90) | 0.58 (0.54-0.63) | 0.73 (0.71-0.75) |
| MPCE Lower middle | 0.78 (0.77-0.80) | 0.27 (0.23-0.31) | 0.94 (0.93-0.95) | 0.59 (0.53-0.65) | 0.76 (0.74-0.78) |
| MPCE Middle | 0.81 (0.80-0.83) | 0.20 (0.17-0.24) | 0.96 (0.95-0.97) | 0.55 (0.47-0.62) | 0.79 (0.76-0.81) |
| MPCE Upper middle | 0.87 (0.86-0.88) | 0.15 (0.11-0.20) | 0.97 (0.97-0.98) | 0.45 (0.35-0.55) | 0.77 (0.74-0.80) |
| MPCE Highest | 0.91 (0.90-0.92) | 0.14 (0.09-0.19) | 0.99 (0.99-0.99) | 0.58 (0.43-0.72) | 0.79 (0.76-0.83) |
| **Gradient Boosting** | | | | | |
| Overall | 0.82 (0.82-0.83) | 0.16 (0.15-0.18) | 0.98 (0.98-0.98) | 0.65 (0.61-0.69) | 0.80 (0.79-0.81) |
| General | 0.88 (0.87-0.89) | 0.11 (0.08-0.14) | 0.99 (0.99-1.00) | 0.71 (0.59-0.82) | 0.80 (0.78-0.82) |
| Scheduled caste | 0.79 (0.77-0.81) | 0.26 (0.22-0.30) | 0.95 (0.94-0.96) | 0.63 (0.56-0.70) | 0.81 (0.79-0.83) |
| Scheduled tribe | 0.78 (0.76-0.80) | 0.17 (0.14-0.21) | 0.98 (0.97-0.98) | 0.69 (0.60-0.77) | 0.79 (0.76-0.81) |
| Other backward class | 0.81 (0.80-0.82) | 0.13 (0.11-0.16) | 0.98 (0.98-0.98) | 0.63 (0.55-0.70) | 0.79 (0.77-0.80) |
| MPCE Lowest | 0.74 (0.72-0.75) | 0.29 (0.25-0.32) | 0.93 (0.92-0.95) | 0.65 (0.60-0.71) | 0.75 (0.73-0.77) |
| MPCE Lower middle | 0.78 (0.76-0.79) | 0.13 (0.11-0.16) | 0.97 (0.97-0.98) | 0.62 (0.53-0.71) | 0.77 (0.75-0.79) |
| MPCE Middle | 0.82 (0.80-0.83) | 0.11 (0.08-0.14) | 0.99 (0.98-0.99) | 0.68 (0.56-0.78) | 0.79 (0.77-0.81) |
| MPCE Upper middle | 0.88 (0.87-0.89) | 0.09 (0.06-0.12) | 0.99 (0.99-0.99) | 0.58 (0.43-0.73) | 0.78 (0.76-0.81) |
| MPCE Highest | 0.91 (0.90-0.93) | 0.08 (0.04-0.12) | 1.00 (1.00-1.00) | 0.79 (0.58-0.95) | 0.81 (0.77-0.84) |
| **LightGBM** | | | | | |
| Overall | 0.82 (0.82-0.83) | 0.22 (0.20-0.23) | 0.97 (0.96-0.97) | 0.60 (0.57-0.63) | 0.80 (0.79-0.81) |
| General | 0.88 (0.87-0.89) | 0.14 (0.10-0.17) | 0.99 (0.98-0.99) | 0.60 (0.49-0.71) | 0.80 (0.78-0.82) |
| Scheduled caste | 0.79 (0.78-0.81) | 0.31 (0.27-0.36) | 0.94 (0.93-0.95) | 0.61 (0.55-0.67) | 0.81 (0.79-0.83) |
| Scheduled tribe | 0.79 (0.77-0.81) | 0.26 (0.22-0.30) | 0.95 (0.94-0.96) | 0.63 (0.56-0.69) | 0.78 (0.76-0.81) |
| Other backward class | 0.81 (0.80-0.82) | 0.18 (0.15-0.21) | 0.97 (0.96-0.97) | 0.57 (0.51-0.63) | 0.79 (0.77-0.80) |
| MPCE Lowest | 0.73 (0.71-0.75) | 0.32 (0.28-0.36) | 0.91 (0.89-0.92) | 0.61 (0.56-0.66) | 0.75 (0.73-0.77) |
| MPCE Lower middle | 0.78 (0.77-0.80) | 0.23 (0.20-0.27) | 0.95 (0.94-0.96) | 0.61 (0.54-0.67) | 0.77 (0.75-0.79) |
| MPCE Middle | 0.81 (0.79-0.83) | 0.16 (0.13-0.20) | 0.97 (0.96-0.98) | 0.55 (0.47-0.64) | 0.79 (0.77-0.81) |
| MPCE Upper middle | 0.88 (0.87-0.89) | 0.14 (0.10-0.18) | 0.98 (0.98-0.99) | 0.57 (0.46-0.68) | 0.79 (0.76-0.82) |
| MPCE Highest | 0.91 (0.90-0.93) | 0.08 (0.04-0.11) | 1.00 (0.99-1.00) | 0.71 (0.50-0.90) | 0.81 (0.78-0.84) |
| **Deep Neural Networks (DNN)** | | | | | |
| Overall | 0.79 (0.79-0.80) | 0.37 (0.35-0.39) | 0.89 (0.89-0.90) | 0.45 (0.43-0.47) | 0.76 (0.75-0.77) |
| General | 0.86 (0.85-0.87) | 0.26 (0.21-0.30) | 0.95 (0.94-0.95) | 0.40 (0.34-0.47) | 0.75 (0.73-0.78) |
| Scheduled caste | 0.76 (0.75-0.78) | 0.48 (0.43-0.53) | 0.85 (0.83-0.87) | 0.50 (0.45-0.54) | 0.78 (0.75-0.80) |
| Scheduled tribe | 0.75 (0.73-0.77) | 0.43 (0.38-0.47) | 0.85 (0.83-0.87) | 0.47 (0.42-0.52) | 0.74 (0.72-0.77) |
| Other backward class | 0.78 (0.76-0.79) | 0.34 (0.30-0.37) | 0.89 (0.87-0.90) | 0.42 (0.38-0.46) | 0.74 (0.72-0.75) |
| MPCE Lowest | 0.68 (0.66-0.70) | 0.48 (0.44-0.52) | 0.77 (0.75-0.79) | 0.48 (0.44-0.52) | 0.70 (0.68-0.72) |
| MPCE Lower middle | 0.76 (0.74-0.78) | 0.40 (0.36-0.45) | 0.87 (0.85-0.88) | 0.48 (0.44-0.53) | 0.74 (0.71-0.76) |
| MPCE Middle | 0.79 (0.77-0.80) | 0.31 (0.27-0.35) | 0.90 (0.89-0.91) | 0.43 (0.38-0.48) | 0.73 (0.71-0.76) |
| MPCE Upper middle | 0.84 (0.83-0.86) | 0.25 (0.20-0.30) | 0.93 (0.92-0.94) | 0.33 (0.27-0.40) | 0.74 (0.72-0.77) |
| MPCE Highest | 0.90 (0.89-0.92) | 0.24 (0.18-0.30) | 0.97 (0.96-0.98) | 0.44 (0.34-0.53) | 0.76 (0.72-0.79) |
| **Fully Convolutional Networks (FCN)** | | | | | |
| Overall | 0.77 (0.76-0.78) | 0.32 (0.30-0.34) | 0.88 (0.87-0.89) | 0.38 (0.36-0.41) | 0.71 (0.70-0.72) |
| General | 0.84 (0.82-0.85) | 0.21 (0.17-0.25) | 0.93 (0.92-0.94) | 0.29 (0.23-0.34) | 0.70 (0.67-0.72) |
| Scheduled caste | 0.74 (0.72-0.75) | 0.39 (0.34-0.43) | 0.84 (0.82-0.86) | 0.43 (0.38-0.47) | 0.72 (0.69-0.74) |
| Scheduled tribe | 0.73 (0.71-0.75) | 0.36 (0.32-0.41) | 0.84 (0.82-0.86) | 0.42 (0.37-0.47) | 0.69 (0.66-0.71) |
| Other backward class | 0.76 (0.75-0.78) | 0.30 (0.27-0.33) | 0.88 (0.87-0.89) | 0.38 (0.35-0.42) | 0.70 (0.68-0.72) |
| MPCE Lowest | 0.66 (0.64-0.68) | 0.39 (0.36-0.43) | 0.78 (0.76-0.80) | 0.44 (0.40-0.48) | 0.66 (0.63-0.68) |
| MPCE Lower middle | 0.74 (0.72-0.75) | 0.33 (0.29-0.37) | 0.86 (0.85-0.88) | 0.42 (0.38-0.47) | 0.68 (0.65-0.70) |
| MPCE Middle | 0.77 (0.75-0.78) | 0.31 (0.27-0.35) | 0.88 (0.86-0.89) | 0.38 (0.33-0.43) | 0.69 (0.66-0.72) |
| MPCE Upper middle | 0.82 (0.80-0.83) | 0.23 (0.18-0.28) | 0.90 (0.89-0.92) | 0.25 (0.20-0.31) | 0.68 (0.65-0.71) |
| MPCE Highest | 0.88 (0.87-0.89) | 0.16 (0.12-0.22) | 0.95 (0.94-0.96) | 0.25 (0.18-0.33) | 0.71 (0.67-0.74) |

1. **Overweight/Obesity**

|  | **Accuracy**  **(95% CI)** | **Sensitivity**  **(95% CI)** | **Specificity**  **(95% CI)** | **Precision**  **(95% CI)** | **AUROC**  **(95% CI)** |
| --- | --- | --- | --- | --- | --- |
| **Logistic Regression** | | | | | |
| Overall | 0.73 (0.72-0.74) | 0.65 (0.64-0.67) | 0.79 (0.78-0.80) | 0.71 (0.69-0.72) | 0.80 (0.79-0.81) |
| General | 0.71 (0.69-0.73) | 0.78 (0.76-0.80) | 0.62 (0.59-0.64) | 0.73 (0.70-0.75) | 0.78 (0.76-0.79) |
| Scheduled caste | 0.75 (0.73-0.77) | 0.45 (0.40-0.48) | 0.89 (0.87-0.90) | 0.64 (0.59-0.68) | 0.78 (0.76-0.80) |
| Scheduled tribe | 0.73 (0.71-0.75) | 0.53 (0.50-0.57) | 0.85 (0.83-0.87) | 0.69 (0.65-0.73) | 0.79 (0.77-0.81) |
| Other backward class | 0.73 (0.72-0.74) | 0.65 (0.63-0.67) | 0.79 (0.77-0.81) | 0.71 (0.69-0.73) | 0.80 (0.78-0.81) |
| MPCE Lowest | 0.78 (0.76-0.79) | 0.34 (0.30-0.38) | 0.93 (0.92-0.95) | 0.65 (0.60-0.70) | 0.78 (0.76-0.81) |
| MPCE Lower middle | 0.72 (0.71-0.74) | 0.49 (0.45-0.52) | 0.86 (0.84-0.88) | 0.67 (0.63-0.71) | 0.77 (0.75-0.79) |
| MPCE Middle | 0.72 (0.71-0.74) | 0.64 (0.61-0.67) | 0.79 (0.76-0.81) | 0.69 (0.66-0.72) | 0.79 (0.77-0.81) |
| MPCE Upper middle | 0.69 (0.67-0.71) | 0.71 (0.69-0.74) | 0.66 (0.63-0.69) | 0.71 (0.68-0.74) | 0.76 (0.74-0.78) |
| MPCE Highest | 0.72 (0.70-0.74) | 0.85 (0.83-0.87) | 0.51 (0.48-0.55) | 0.74 (0.72-0.76) | 0.77 (0.75-0.79) |
| **Random Forest** | | | | | |
| Overall | 0.72 (0.71-0.73) | 0.63 (0.62-0.64) | 0.79 (0.78-0.80) | 0.70 (0.69-0.71) | 0.79 (0.78-0.79) |
| General | 0.70 (0.68-0.71) | 0.74 (0.72-0.76) | 0.65 (0.62-0.67) | 0.73 (0.71-0.75) | 0.76 (0.74-0.78) |
| Scheduled caste | 0.74 (0.72-0.76) | 0.44 (0.40-0.47) | 0.87 (0.85-0.89) | 0.61 (0.56-0.65) | 0.76 (0.74-0.79) |
| Scheduled tribe | 0.73 (0.71-0.75) | 0.55 (0.52-0.59) | 0.84 (0.82-0.86) | 0.68 (0.65-0.72) | 0.78 (0.76-0.80) |
| Other backward class | 0.72 (0.71-0.73) | 0.63 (0.61-0.65) | 0.79 (0.78-0.81) | 0.70 (0.68-0.73) | 0.78 (0.77-0.80) |
| MPCE Lowest | 0.77 (0.75-0.79) | 0.33 (0.29-0.37) | 0.93 (0.92-0.95) | 0.64 (0.59-0.69) | 0.77 (0.74-0.79) |
| MPCE Lower middle | 0.72 (0.70-0.74) | 0.48 (0.45-0.52) | 0.86 (0.84-0.87) | 0.66 (0.62-0.69) | 0.75 (0.73-0.77) |
| MPCE Middle | 0.71 (0.69-0.73) | 0.61 (0.58-0.64) | 0.79 (0.76-0.81) | 0.68 (0.65-0.71) | 0.78 (0.76-0.79) |
| MPCE Upper middle | 0.69 (0.67-0.71) | 0.69 (0.67-0.72) | 0.69 (0.66-0.71) | 0.72 (0.70-0.75) | 0.75 (0.73-0.77) |
| MPCE Highest | 0.70 (0.68-0.72) | 0.81 (0.79-0.83) | 0.52 (0.49-0.55) | 0.73 (0.71-0.76) | 0.74 (0.72-0.76) |
| **XGBoost** | | | | | |
| Overall | 0.72 (0.71-0.73) | 0.65 (0.64-0.67) | 0.78 (0.76-0.79) | 0.70 (0.68-0.71) | 0.79 (0.78-0.80) |
| General | 0.70 (0.68-0.71) | 0.77 (0.75-0.79) | 0.61 (0.58-0.64) | 0.72 (0.70-0.74) | 0.77 (0.75-0.78) |
| Scheduled caste | 0.74 (0.72-0.76) | 0.48 (0.44-0.52) | 0.86 (0.84-0.88) | 0.61 (0.56-0.65) | 0.77 (0.75-0.79) |
| Scheduled tribe | 0.72 (0.70-0.74) | 0.53 (0.50-0.57) | 0.83 (0.81-0.85) | 0.66 (0.62-0.70) | 0.78 (0.76-0.80) |
| Other backward class | 0.73 (0.72-0.74) | 0.65 (0.63-0.67) | 0.79 (0.77-0.81) | 0.71 (0.69-0.73) | 0.79 (0.78-0.80) |
| MPCE Lowest | 0.77 (0.75-0.79) | 0.38 (0.34-0.42) | 0.91 (0.90-0.92) | 0.61 (0.56-0.65) | 0.78 (0.76-0.80) |
| MPCE Lower middle | 0.73 (0.71-0.74) | 0.52 (0.48-0.55) | 0.85 (0.83-0.86) | 0.66 (0.62-0.70) | 0.76 (0.74-0.78) |
| MPCE Middle | 0.72 (0.70-0.74) | 0.63 (0.60-0.66) | 0.79 (0.76-0.81) | 0.68 (0.65-0.71) | 0.78 (0.76-0.80) |
| MPCE Upper middle | 0.69 (0.67-0.71) | 0.71 (0.69-0.74) | 0.66 (0.63-0.69) | 0.71 (0.68-0.73) | 0.76 (0.74-0.78) |
| MPCE Highest | 0.70 (0.68-0.72) | 0.82 (0.80-0.84) | 0.52 (0.48-0.55) | 0.73 (0.71-0.76) | 0.75 (0.73-0.77) |
| **Gradient Boosting** | | | | | |
| Overall | 0.72 (0.71-0.73) | 0.64 (0.62-0.65) | 0.79 (0.78-0.80) | 0.71 (0.69-0.72) | 0.79 (0.79-0.80) |
| General | 0.70 (0.69-0.72) | 0.74 (0.73-0.76) | 0.65 (0.62-0.68) | 0.74 (0.71-0.76) | 0.77 (0.75-0.79) |
| Scheduled caste | 0.74 (0.73-0.76) | 0.45 (0.41-0.48) | 0.88 (0.86-0.90) | 0.63 (0.58-0.67) | 0.78 (0.76-0.80) |
| Scheduled tribe | 0.73 (0.71-0.74) | 0.53 (0.50-0.57) | 0.84 (0.82-0.86) | 0.68 (0.64-0.71) | 0.79 (0.77-0.81) |
| Other backward class | 0.72 (0.71-0.74) | 0.64 (0.62-0.66) | 0.79 (0.78-0.81) | 0.71 (0.68-0.73) | 0.79 (0.78-0.80) |
| MPCE Lowest | 0.77 (0.75-0.79) | 0.34 (0.30-0.38) | 0.93 (0.91-0.94) | 0.63 (0.58-0.68) | 0.78 (0.76-0.80) |
| MPCE Lower middle | 0.72 (0.70-0.73) | 0.47 (0.44-0.51) | 0.85 (0.84-0.87) | 0.65 (0.61-0.69) | 0.77 (0.75-0.79) |
| MPCE Middle | 0.72 (0.70-0.74) | 0.62 (0.59-0.65) | 0.79 (0.77-0.81) | 0.69 (0.66-0.72) | 0.78 (0.76-0.80) |
| MPCE Upper middle | 0.69 (0.67-0.71) | 0.69 (0.66-0.72) | 0.69 (0.66-0.72) | 0.72 (0.70-0.74) | 0.76 (0.74-0.78) |
| MPCE Highest | 0.72 (0.70-0.74) | 0.83 (0.81-0.85) | 0.54 (0.51-0.57) | 0.75 (0.72-0.77) | 0.76 (0.74-0.78) |
| **LightGBM** | | | | | |
| Overall | 0.72 (0.71-0.73) | 0.65 (0.64-0.67) | 0.78 (0.77-0.79) | 0.70 (0.68-0.71) | 0.80 (0.79-0.81) |
| General | 0.70 (0.68-0.71) | 0.76 (0.74-0.78) | 0.61 (0.59-0.64) | 0.72 (0.70-0.74) | 0.77 (0.76-0.79) |
| Scheduled caste | 0.74 (0.72-0.76) | 0.47 (0.43-0.51) | 0.87 (0.85-0.89) | 0.62 (0.57-0.66) | 0.79 (0.77-0.81) |
| Scheduled tribe | 0.73 (0.71-0.75) | 0.55 (0.51-0.58) | 0.84 (0.82-0.86) | 0.68 (0.64-0.71) | 0.79 (0.77-0.81) |
| Other backward class | 0.73 (0.72-0.74) | 0.66 (0.64-0.68) | 0.78 (0.77-0.80) | 0.70 (0.68-0.73) | 0.80 (0.78-0.81) |
| MPCE Lowest | 0.78 (0.76-0.79) | 0.37 (0.33-0.41) | 0.92 (0.91-0.94) | 0.63 (0.58-0.69) | 0.78 (0.76-0.81) |
| MPCE Lower middle | 0.72 (0.70-0.74) | 0.52 (0.48-0.55) | 0.84 (0.82-0.86) | 0.65 (0.61-0.68) | 0.76 (0.75-0.79) |
| MPCE Middle | 0.71 (0.69-0.73) | 0.63 (0.60-0.66) | 0.78 (0.75-0.80) | 0.67 (0.65-0.70) | 0.78 (0.76-0.80) |
| MPCE Upper middle | 0.69 (0.67-0.71) | 0.71 (0.68-0.73) | 0.67 (0.65-0.70) | 0.72 (0.69-0.74) | 0.76 (0.74-0.78) |
| MPCE Highest | 0.71 (0.69-0.73) | 0.84 (0.82-0.86) | 0.51 (0.47-0.54) | 0.73 (0.71-0.76) | 0.77 (0.75-0.79) |
| **Deep Neural Networks (DNN)** | | | | | |
| Overall | 0.70 (0.69-0.71) | 0.68 (0.66-0.69) | 0.71 (0.70-0.72) | 0.65 (0.64-0.66) | 0.76 (0.76-0.77) |
| General | 0.68 (0.67-0.70) | 0.78 (0.76-0.80) | 0.56 (0.54-0.59) | 0.70 (0.68-0.72) | 0.74 (0.72-0.76) |
| Scheduled caste | 0.72 (0.70-0.74) | 0.51 (0.47-0.55) | 0.81 (0.79-0.83) | 0.55 (0.51-0.59) | 0.75 (0.73-0.77) |
| Scheduled tribe | 0.70 (0.68-0.72) | 0.58 (0.54-0.61) | 0.77 (0.74-0.79) | 0.61 (0.57-0.64) | 0.75 (0.73-0.77) |
| Other backward class | 0.70 (0.68-0.71) | 0.67 (0.65-0.69) | 0.71 (0.70-0.73) | 0.65 (0.62-0.67) | 0.76 (0.75-0.78) |
| MPCE Lowest | 0.75 (0.73-0.77) | 0.44 (0.40-0.48) | 0.86 (0.85-0.88) | 0.54 (0.50-0.58) | 0.75 (0.72-0.77) |
| MPCE Lower middle | 0.69 (0.67-0.71) | 0.56 (0.53-0.59) | 0.76 (0.74-0.79) | 0.58 (0.54-0.61) | 0.73 (0.71-0.75) |
| MPCE Middle | 0.68 (0.66-0.70) | 0.65 (0.63-0.68) | 0.70 (0.68-0.73) | 0.62 (0.59-0.65) | 0.75 (0.73-0.77) |
| MPCE Upper middle | 0.67 (0.65-0.69) | 0.73 (0.71-0.76) | 0.60 (0.56-0.63) | 0.68 (0.65-0.70) | 0.74 (0.72-0.76) |
| MPCE Highest | 0.69 (0.67-0.71) | 0.82 (0.80-0.84) | 0.49 (0.46-0.53) | 0.72 (0.70-0.75) | 0.73 (0.70-0.75) |
| **Fully Convolutional Networks (FCN)** | | | | | |
| Overall | 0.66 (0.65-0.67) | 0.63 (0.61-0.64) | 0.68 (0.67-0.69) | 0.61 (0.59-0.62) | 0.71 (0.70-0.72) |
| General | 0.66 (0.64-0.67) | 0.73 (0.71-0.75) | 0.57 (0.54-0.59) | 0.69 (0.66-0.71) | 0.70 (0.68-0.72) |
| Scheduled caste | 0.67 (0.65-0.69) | 0.47 (0.43-0.50) | 0.76 (0.74-0.79) | 0.47 (0.43-0.51) | 0.68 (0.66-0.71) |
| Scheduled tribe | 0.66 (0.64-0.68) | 0.54 (0.50-0.58) | 0.73 (0.71-0.76) | 0.55 (0.52-0.59) | 0.68 (0.66-0.71) |
| Other backward class | 0.65 (0.64-0.67) | 0.62 (0.60-0.64) | 0.67 (0.65-0.69) | 0.60 (0.58-0.62) | 0.70 (0.68-0.72) |
| MPCE Lowest | 0.71 (0.69-0.72) | 0.44 (0.41-0.48) | 0.80 (0.78-0.82) | 0.45 (0.40-0.48) | 0.69 (0.66-0.72) |
| MPCE Lower middle | 0.66 (0.64-0.68) | 0.54 (0.51-0.58) | 0.74 (0.71-0.76) | 0.54 (0.51-0.57) | 0.68 (0.66-0.71) |
| MPCE Middle | 0.64 (0.62-0.66) | 0.59 (0.56-0.62) | 0.67 (0.65-0.70) | 0.57 (0.54-0.60) | 0.68 (0.66-0.70) |
| MPCE Upper middle | 0.62 (0.61-0.64) | 0.65 (0.63-0.68) | 0.59 (0.56-0.62) | 0.65 (0.62-0.68) | 0.67 (0.65-0.69) |
| MPCE Highest | 0.66 (0.64-0.68) | 0.77 (0.74-0.79) | 0.48 (0.45-0.52) | 0.71 (0.68-0.73) | 0.69 (0.66-0.71) |

1. **High Waist** **Circumference**

|  | **Accuracy**  **(95% CI)** | **Sensitivity**  **(95% CI)** | **Specificity**  **(95% CI)** | **Precision**  **(95% CI)** | **AUROC**  **(95% CI)** |
| --- | --- | --- | --- | --- | --- |
| **Logistic Regression** | | | | | |
| Overall | 0.75 (0.74-0.76) | 0.71 (0.70-0.72) | 0.79 (0.78-0.80) | 0.74 (0.73-0.75) | 0.84 (0.83-0.84) |
| General | 0.75 (0.74-0.77) | 0.81 (0.79-0.83) | 0.66 (0.63-0.69) | 0.77 (0.76-0.79) | 0.83 (0.82-0.85) |
| Scheduled caste | 0.77 (0.75-0.79) | 0.51 (0.47-0.55) | 0.89 (0.87-0.90) | 0.67 (0.63-0.71) | 0.82 (0.80-0.84) |
| Scheduled tribe | 0.74 (0.72-0.76) | 0.64 (0.61-0.67) | 0.83 (0.80-0.85) | 0.74 (0.70-0.77) | 0.83 (0.81-0.85) |
| Other backward class | 0.75 (0.73-0.76) | 0.71 (0.69-0.73) | 0.78 (0.76-0.79) | 0.72 (0.70-0.74) | 0.83 (0.82-0.84) |
| MPCE Lowest | 0.77 (0.75-0.79) | 0.51 (0.47-0.55) | 0.89 (0.87-0.90) | 0.67 (0.63-0.72) | 0.82 (0.80-0.83) |
| MPCE Lower middle | 0.75 (0.73-0.76) | 0.62 (0.59-0.65) | 0.83 (0.81-0.85) | 0.69 (0.66-0.72) | 0.82 (0.80-0.83) |
| MPCE Middle | 0.75 (0.73-0.76) | 0.72 (0.69-0.75) | 0.77 (0.74-0.79) | 0.72 (0.69-0.74) | 0.84 (0.82-0.85) |
| MPCE Upper middle | 0.75 (0.73-0.77) | 0.75 (0.72-0.77) | 0.75 (0.72-0.77) | 0.78 (0.76-0.81) | 0.83 (0.81-0.84) |
| MPCE Highest | 0.74 (0.72-0.76) | 0.83 (0.81-0.85) | 0.61 (0.58-0.64) | 0.77 (0.74-0.79) | 0.83 (0.81-0.85) |
| **Random Forest** | | | | | |
| Overall | 0.75 (0.74-0.76) | 0.69 (0.68-0.70) | 0.80 (0.79-0.81) | 0.75 (0.73-0.76) | 0.83 (0.82-0.84) |
| General | 0.75 (0.73-0.77) | 0.78 (0.76-0.80) | 0.71 (0.69-0.74) | 0.79 (0.78-0.81) | 0.83 (0.81-0.84) |
| Scheduled caste | 0.77 (0.75-0.79) | 0.52 (0.48-0.56) | 0.89 (0.87-0.90) | 0.67 (0.63-0.71) | 0.82 (0.80-0.84) |
| Scheduled tribe | 0.74 (0.73-0.76) | 0.64 (0.60-0.67) | 0.83 (0.81-0.85) | 0.74 (0.71-0.77) | 0.82 (0.80-0.84) |
| Other backward class | 0.74 (0.73-0.75) | 0.68 (0.66-0.70) | 0.79 (0.77-0.80) | 0.72 (0.70-0.74) | 0.82 (0.80-0.83) |
| MPCE Lowest | 0.77 (0.75-0.79) | 0.50 (0.46-0.53) | 0.89 (0.88-0.91) | 0.67 (0.63-0.71) | 0.81 (0.79-0.83) |
| MPCE Lower middle | 0.74 (0.73-0.76) | 0.61 (0.58-0.65) | 0.83 (0.81-0.85) | 0.69 (0.66-0.72) | 0.81 (0.79-0.82) |
| MPCE Middle | 0.74 (0.72-0.76) | 0.68 (0.65-0.71) | 0.79 (0.77-0.81) | 0.73 (0.70-0.75) | 0.83 (0.81-0.84) |
| MPCE Upper middle | 0.74 (0.72-0.76) | 0.72 (0.69-0.74) | 0.76 (0.74-0.79) | 0.79 (0.76-0.81) | 0.82 (0.80-0.84) |
| MPCE Highest | 0.75 (0.73-0.77) | 0.81 (0.79-0.83) | 0.66 (0.63-0.69) | 0.78 (0.76-0.81) | 0.82 (0.80-0.84) |
| **XGBoost** | | | | | |
| Overall | 0.75 (0.74-0.76) | 0.71 (0.69-0.72) | 0.79 (0.78-0.80) | 0.74 (0.73-0.75) | 0.83 (0.82-0.84) |
| General | 0.75 (0.74-0.77) | 0.80 (0.78-0.82) | 0.68 (0.65-0.71) | 0.78 (0.76-0.80) | 0.83 (0.82-0.84) |
| Scheduled caste | 0.76 (0.75-0.78) | 0.52 (0.48-0.56) | 0.88 (0.86-0.89) | 0.65 (0.61-0.69) | 0.81 (0.79-0.83) |
| Scheduled tribe | 0.74 (0.72-0.76) | 0.64 (0.61-0.68) | 0.82 (0.80-0.84) | 0.73 (0.69-0.76) | 0.82 (0.81-0.84) |
| Other backward class | 0.74 (0.73-0.76) | 0.70 (0.68-0.72) | 0.78 (0.76-0.79) | 0.72 (0.70-0.74) | 0.82 (0.81-0.83) |
| MPCE Lowest | 0.78 (0.76-0.80) | 0.54 (0.51-0.58) | 0.88 (0.87-0.90) | 0.68 (0.64-0.72) | 0.82 (0.80-0.84) |
| MPCE Lower middle | 0.74 (0.73-0.76) | 0.62 (0.59-0.66) | 0.82 (0.80-0.84) | 0.69 (0.65-0.72) | 0.81 (0.79-0.83) |
| MPCE Middle | 0.73 (0.72-0.75) | 0.69 (0.66-0.72) | 0.77 (0.74-0.79) | 0.71 (0.68-0.74) | 0.82 (0.81-0.84) |
| MPCE Upper middle | 0.74 (0.72-0.75) | 0.74 (0.71-0.76) | 0.74 (0.71-0.76) | 0.77 (0.75-0.80) | 0.82 (0.81-0.84) |
| MPCE Highest | 0.75 (0.73-0.77) | 0.82 (0.80-0.84) | 0.64 (0.61-0.67) | 0.78 (0.76-0.80) | 0.82 (0.80-0.84) |
| **Gradient Boosting** | | | | | |
| Overall | 0.75 (0.75-0.76) | 0.70 (0.69-0.72) | 0.80 (0.79-0.81) | 0.75 (0.73-0.76) | 0.84 (0.83-0.84) |
| General | 0.75 (0.74-0.77) | 0.78 (0.76-0.81) | 0.71 (0.68-0.73) | 0.79 (0.77-0.81) | 0.83 (0.82-0.85) |
| Scheduled caste | 0.77 (0.76-0.79) | 0.53 (0.49-0.57) | 0.88 (0.87-0.90) | 0.67 (0.63-0.71) | 0.82 (0.80-0.84) |
| Scheduled tribe | 0.75 (0.73-0.77) | 0.66 (0.63-0.70) | 0.82 (0.79-0.84) | 0.73 (0.70-0.76) | 0.83 (0.81-0.85) |
| Other backward class | 0.74 (0.73-0.76) | 0.70 (0.68-0.72) | 0.78 (0.76-0.80) | 0.72 (0.70-0.74) | 0.82 (0.81-0.84) |
| MPCE Lowest | 0.77 (0.76-0.79) | 0.49 (0.45-0.53) | 0.90 (0.88-0.92) | 0.69 (0.65-0.73) | 0.81 (0.80-0.83) |
| MPCE Lower middle | 0.75 (0.73-0.77) | 0.64 (0.61-0.68) | 0.81 (0.79-0.84) | 0.69 (0.65-0.71) | 0.82 (0.80-0.83) |
| MPCE Middle | 0.75 (0.73-0.77) | 0.71 (0.68-0.73) | 0.78 (0.76-0.81) | 0.73 (0.70-0.76) | 0.83 (0.82-0.85) |
| MPCE Upper middle | 0.75 (0.73-0.76) | 0.73 (0.71-0.76) | 0.76 (0.74-0.79) | 0.79 (0.77-0.82) | 0.83 (0.81-0.84) |
| MPCE Highest | 0.75 (0.73-0.77) | 0.82 (0.80-0.84) | 0.64 (0.60-0.67) | 0.78 (0.75-0.80) | 0.83 (0.81-0.84) |
| **LightGBM** | | | | | |
| Overall | 0.75 (0.75-0.76) | 0.71 (0.70-0.72) | 0.79 (0.78-0.80) | 0.75 (0.73-0.76) | 0.84 (0.83-0.85) |
| General | 0.75 (0.74-0.77) | 0.80 (0.78-0.81) | 0.69 (0.67-0.72) | 0.79 (0.77-0.80) | 0.83 (0.82-0.85) |
| Scheduled caste | 0.77 (0.75-0.79) | 0.53 (0.49-0.56) | 0.88 (0.86-0.90) | 0.66 (0.62-0.70) | 0.83 (0.81-0.85) |
| Scheduled tribe | 0.76 (0.74-0.78) | 0.66 (0.63-0.69) | 0.84 (0.82-0.86) | 0.76 (0.72-0.79) | 0.83 (0.81-0.85) |
| Other backward class | 0.75 (0.73-0.76) | 0.71 (0.68-0.73) | 0.78 (0.76-0.80) | 0.72 (0.70-0.74) | 0.83 (0.82-0.84) |
| MPCE Lowest | 0.78 (0.76-0.79) | 0.52 (0.48-0.56) | 0.89 (0.88-0.91) | 0.68 (0.64-0.73) | 0.82 (0.80-0.84) |
| MPCE Lower middle | 0.75 (0.74-0.77) | 0.64 (0.61-0.67) | 0.82 (0.80-0.84) | 0.70 (0.67-0.73) | 0.82 (0.80-0.84) |
| MPCE Middle | 0.75 (0.73-0.76) | 0.70 (0.68-0.73) | 0.78 (0.76-0.80) | 0.72 (0.70-0.75) | 0.83 (0.82-0.85) |
| MPCE Upper middle | 0.75 (0.73-0.76) | 0.74 (0.71-0.76) | 0.76 (0.73-0.78) | 0.79 (0.76-0.81) | 0.83 (0.81-0.85) |
| MPCE Highest | 0.75 (0.73-0.77) | 0.83 (0.80-0.85) | 0.63 (0.60-0.66) | 0.78 (0.75-0.80) | 0.83 (0.81-0.85) |
| **Deep Neural Networks (DNN)** | | | | | |
| Overall | 0.73 (0.73-0.74) | 0.68 (0.67-0.69) | 0.78 (0.77-0.79) | 0.73 (0.71-0.74) | 0.81 (0.80-0.82) |
| General | 0.73 (0.72-0.75) | 0.77 (0.75-0.79) | 0.67 (0.65-0.70) | 0.77 (0.75-0.79) | 0.80 (0.79-0.82) |
| Scheduled caste | 0.76 (0.74-0.78) | 0.52 (0.48-0.56) | 0.87 (0.85-0.89) | 0.64 (0.60-0.69) | 0.80 (0.78-0.82) |
| Scheduled tribe | 0.73 (0.71-0.75) | 0.61 (0.58-0.65) | 0.81 (0.79-0.83) | 0.71 (0.67-0.74) | 0.80 (0.78-0.82) |
| Other backward class | 0.73 (0.71-0.74) | 0.67 (0.65-0.69) | 0.77 (0.76-0.79) | 0.71 (0.68-0.73) | 0.79 (0.78-0.81) |
| MPCE Lowest | 0.75 (0.74-0.77) | 0.55 (0.51-0.59) | 0.85 (0.83-0.87) | 0.62 (0.58-0.66) | 0.79 (0.77-0.81) |
| MPCE Lower middle | 0.73 (0.71-0.75) | 0.58 (0.55-0.62) | 0.82 (0.80-0.84) | 0.67 (0.64-0.70) | 0.79 (0.77-0.81) |
| MPCE Middle | 0.73 (0.71-0.75) | 0.67 (0.64-0.70) | 0.78 (0.75-0.80) | 0.71 (0.68-0.74) | 0.81 (0.79-0.82) |
| MPCE Upper middle | 0.72 (0.70-0.74) | 0.72 (0.69-0.74) | 0.73 (0.70-0.76) | 0.76 (0.74-0.79) | 0.80 (0.78-0.82) |
| MPCE Highest | 0.74 (0.72-0.76) | 0.78 (0.76-0.80) | 0.67 (0.64-0.70) | 0.78 (0.76-0.80) | 0.80 (0.78-0.82) |
| **Fully Convolutional Networks (FCN)** | | | | | |
| Overall | 0.69 (0.68-0.69) | 0.67 (0.66-0.69) | 0.70 (0.68-0.71) | 0.65 (0.64-0.67) | 0.76 (0.75-0.76) |
| General | 0.69 (0.67-0.71) | 0.75 (0.73-0.77) | 0.61 (0.58-0.64) | 0.73 (0.71-0.75) | 0.75 (0.73-0.77) |
| Scheduled caste | 0.71 (0.69-0.73) | 0.53 (0.50-0.57) | 0.79 (0.77-0.81) | 0.53 (0.49-0.57) | 0.74 (0.72-0.77) |
| Scheduled tribe | 0.68 (0.66-0.70) | 0.64 (0.60-0.67) | 0.71 (0.68-0.73) | 0.62 (0.59-0.66) | 0.74 (0.71-0.76) |
| Other backward class | 0.67 (0.66-0.69) | 0.67 (0.65-0.69) | 0.68 (0.66-0.70) | 0.63 (0.61-0.65) | 0.74 (0.73-0.76) |
| MPCE Lowest | 0.71 (0.69-0.72) | 0.56 (0.52-0.59) | 0.77 (0.75-0.79) | 0.52 (0.49-0.56) | 0.75 (0.72-0.77) |
| MPCE Lower middle | 0.67 (0.65-0.69) | 0.61 (0.58-0.64) | 0.71 (0.69-0.74) | 0.57 (0.54-0.60) | 0.73 (0.71-0.75) |
| MPCE Middle | 0.68 (0.67-0.70) | 0.67 (0.64-0.70) | 0.70 (0.67-0.72) | 0.64 (0.61-0.67) | 0.75 (0.73-0.77) |
| MPCE Upper middle | 0.69 (0.67-0.71) | 0.70 (0.68-0.73) | 0.67 (0.64-0.70) | 0.72 (0.70-0.75) | 0.75 (0.73-0.77) |
| MPCE Highest | 0.68 (0.66-0.70) | 0.76 (0.73-0.78) | 0.56 (0.53-0.59) | 0.73 (0.70-0.75) | 0.73 (0.71-0.75) |
