# Supplementary material for: Evaluating algorithmic fairness of machine learning models in predicting underweight, overweight, and adiposity across socioeconomic and caste groups in India: evidence from the longitudinal ageing study in India
Source: PLOS Digit Health. 2025 Nov 26;4(11):e0000951. doi: 10.1371/journal.pdig.0000951 (PMC12654920; doi:10.1371/journal.pdig.0000951)
Supplement: S4 Table — (DOCX) [file pdig.0000951.s004.docx]

**S4 Table. Performance of the Subgroup-Stratified models**

1. **Underweight**

|  | **Before(Using LightGBM)** | | | **Subgroup Best Model** | | | |
| --- | --- | --- | --- | --- | --- | --- | --- |
|  | **AUROC** | **Sensitivity** | **Specificity** | **Model** | **AUROC** | **Sensitivity** | **Specificity** |
| **Caste** |  |  |  |  |  |  |  |
| General | 0.8021 | 0.1384 | 0.9867 | Logistic Regression | 0.8038 | 0.1834 | 0.9773 |
| Scheduled caste | 0.8124 | 0.3126 | 0.9391 | Logistic Regression | 0.7986 | 0.3486 | 0.9121 |
| Scheduled tribe | 0.7841 | 0.2641 | 0.9510 | Gradient Boosting | 0.7442 | 0.1639 | 0.9559 |
| Other backward class | 0.7883 | 0.1791 | 0.9664 | Logistic Regression | 0.7925 | 0.1997 | 0.9682 |
| **MPCE** |  |  |  |  |  |  |  |
| MPCE Lowest | 0.7489 | 0.3204 | 0.9094 | Logistic Regression | 0.7445 | 0.3200 | 0.9147 |
| MPCE Lower middle | 0.7705 | 0.2308 | 0.9545 | Logistic Regression | 0.7784 | 0.2956 | 0.9462 |
| MPCE Middle | 0.7918 | 0.1648 | 0.9677 | Logistic Regression | 0.7984 | 0.1859 | 0.9808 |
| MPCE Upper middle | 0.7887 | 0.1368 | 0.9850 | Gradient Boosting | 0.7740 | 0.0979 | 0.9859 |
| MPCE Highest | 0.8119 | 0.0777 | 0.9969 | Gradient Boosting | 0.8114 | 0.0553 | 0.9929 |

1. **Overweight/Obesity**

|  | **Before** | | | **Subgroup Best Model** | | | |
| --- | --- | --- | --- | --- | --- | --- | --- |
|  | **AUROC** | **Sensitivity** | **Specificity** | **Model** | **AUROC** | **Sensitivity** | **Specificity** |
| **Caste** |  |  |  |  |  |  |  |
| General | 0.7734 | 0.7611 | 0.6143 | Logistic Regression | 0.7679 | 0.7775 | 0.6086 |
| Scheduled caste | 0.7851 | 0.4703 | 0.8681 | Gradient Boosting | 0.7861 | 0.4351 | 0.8956 |
| Scheduled tribe | 0.7886 | 0.5489 | 0.8394 | Logistic Regression | 0.7743 | 0.5121 | 0.8506 |
| Other backward class | 0.7966 | 0.6576 | 0.7843 | Logistic Regression | 0.7790 | 0.6400 | 0.7624 |
| **MPCE** |  |  |  |  |  |  |  |
| MPCE Lowest | 0.7822 | 0.3694 | 0.9225 | Logistic Regression | 0.7725 | 0.3587 | 0.9370 |
| MPCE Lower middle | 0.7648 | 0.5171 | 0.8383 | Logistic Regression | 0.7573 | 0.4883 | 0.8490 |
| MPCE Middle | 0.7843 | 0.6289 | 0.7763 | Logistic Regression | 0.7561 | 0.6077 | 0.7682 |
| MPCE Upper middle | 0.7645 | 0.7066 | 0.6742 | Logistic Regression | 0.7698 | 0.7372 | 0.6764 |
| MPCE Highest | 0.7674 | 0.837633 | 0.5098 | Logistic Regression | 0.7624 | 0.8278 | 0.5445 |

1. **High Waist Circumference**

|  | **Before** | | | **Subgroup Best Model** | | | |
| --- | --- | --- | --- | --- | --- | --- | --- |
|  | **AUROC** | **Sensitivity** | **Specificity** | **Model** | **AUROC** | **Sensitivity** | **Specificity** |
| **Caste** |  |  |  |  |  |  |  |
| General | 0.8332 | 0.7953 | 0.6910 | Logistic Regression | 0.8300 | 0.8169 | 0.6696 |
| Scheduled caste | 0.8268 | 0.5267 | 0.8786 | Gradient Boosting | 0.8366 | 0.5270 | 0.9019 |
| Scheduled tribe | 0.8298 | 0.6600 | 0.8382 | Logistic Regression | 0.8167 | 0.6654 | 0.8029 |
| Other backward class | 0.8291 | 0.7055 | 0.7794 | Logistic Regression | 0.8141 | 0.6967 | 0.7580 |
| **MPCE** |  |  |  |  |  |  |  |
| MPCE Lowest | 0.8210 | 0.5192 | 0.8925 | Logistic Regression | 0.8326 | 0.5365 | 0.8937 |
| MPCE Lower middle | 0.8204 | 0.6433 | 0.8246 | Logistic Regression | 0.8128 | 0.6459 | 0.8230 |
| MPCE Middle | 0.8335 | 0.7048 | 0.7790 | Logistic Regression | 0.8121 | 0.7019 | 0.7615 |
| MPCE Upper middle | 0.8296 | 0.7373 | 0.7556 | Logistic Regression | 0.8133 | 0.7600 | 0.7043 |
| MPCE Highest | 0.8299 | 0.8270 | 0.6335 | Gradient Boosting | 0.8226 | 0.8164 | 0.6390 |
