# Supplementary material for: Evaluating algorithmic fairness of machine learning models in predicting underweight, overweight, and adiposity across socioeconomic and caste groups in India: evidence from the longitudinal ageing study in India
Source: PLOS Digit Health. 2025 Nov 26;4(11):e0000951. doi: 10.1371/journal.pdig.0000951 (PMC12654920; doi:10.1371/journal.pdig.0000951)
Supplement: S5 Table — (DOCX) [file pdig.0000951.s005.docx]

**S5 Table. Comparison of Fairness Metrics (Equalized odds) Among Different Bias Mitigation Methods**

|  | 1. **Underweight** | 1. **Overweight/Obesity** | 1. **High Waist Circumference** |
| --- | --- | --- | --- |
| **Basic** |  |  |  |
| Caste | 0.0601 | 0.1382 | 0.1334 |
| MPCE | 0.0863 | 0.2246 | 0.1441 |
| **Disparate Impact Remover** |  |  |  |
| Caste | 0.0601 | 0.1523 | 0.1167 |
| MPCE | 0.0849 | 0.2099 | 0.1370 |
| **Reweighing** |  |  |  |
| Caste | 0.0606 | 0.1419 | 0.1140 |
| MPCE | 0.0790 | 0.1982 | 0.1230 |
| **Prejudice Remover** |  |  |  |
| Caste | 0.0121 | 0.1607 | 0.1741 |
| MPCE | 0.1002 | 0.1023 | 0.0798 |
| **Exponentiated Gradient Reduction** |  |  |  |
| Caste | 0.0310 | 0.0355 | 0.0280 |
| MPCE | 0.0143 | 0.0204 | 0.0326 |
| **Adversarial Debiasing** |  |  |  |
| Caste | 0.0015 | 0.0771 | 0.0167 |
| MPCE | 0.0022 | 0.1278 | 0.0351 |
| **Reject Option Classification** |  |  |  |
| Caste | 0.0424 | 0.0661 | 0.0415 |
| MPCE | 0.0446 | 0.0947 | 0.0476 |
| **Equalized Odds Postprocessing** |  |  |  |
| Caste | 0.0183 | 0.0617 | 0.0515 |
| MPCE | 0.0173 | 0.0701 | 0.0380 |
| **Stratified Subgroup Best Model** |  |  |  |
| Caste | 0.0915 | 0.1696 | 0.1449 |
| MPCE | 0.0955 | 0.2285 | 0.1360 |
