# Supplementary material for: Evaluating algorithmic fairness of machine learning models in predicting underweight, overweight, and adiposity across socioeconomic and caste groups in India: evidence from the longitudinal ageing study in India
Source: PLOS Digit Health. 2025 Nov 26;4(11):e0000951. doi: 10.1371/journal.pdig.0000951 (PMC12654920; doi:10.1371/journal.pdig.0000951)
Supplement: Attachment — Submitted filename: Response to Reviewers.pdf [file pdig.0000951.s007.pdf]

Reviewer #1: This is very timely research and advances the field by identifying the gaps in ML technique application in a healthcare context. My expertise is in the health domain, and I have reviewed the manuscript from a healthcare application perspective. I would be relying on ML expert reviewers on the robustness of the ML application. From a healthcare application perspective, there are two key components that I would like to see in the paper to make it more relevant and informative for healthcare audiences:

Clarify criteria for subgroup selection: Please elaborate on the rationale for identifying and selecting certain groups as marginalized sub-populations. Explicitly stating the basis for these choices will help readers understand the context and strengthen the justification for your approach.

Thank you for your question and suggestion. We selected caste (Scheduled Caste, Scheduled Tribe, Other Backward Classes, and General) and household economic status (MPCE) because they are legally recognized axes of structural disadvantage in India and are consistently associated with large, persistent gaps in health outcomes, healthcare use, and financial risk. National studies document pronounced caste- and wealth-related inequalities in NCDs, nutrition, mortality, and service coverage; MPCE is the Government of India's standard indicator of living standards. Auditing ML performance and bias across these socially salient subgroups aligns with global guidance on equity in AI for health. Additionally, we have added this rationale and associated literatures to the Method section.

Provide real-world implementation examples: Could you expand on where these machine learning techniques have been applied in real-world settings? Including concrete examples, particularly in healthcare contexts, would help readers better appreciate the relevance and potential impact of your work.

Thank you for the suggestion. We now add a short note and literatures in Methods situating our models in practice: tree-ensemble approaches are commonly implemented for EHR-based risk stratification (e.g., early-warning systems) and adverse-event prediction, as well as chronic-disease risk modeling; deep neural networks are widely used in imaging workflows, including diabetic-retinopathy screening and computer-aided detection for TB chest-X-ray screening.

Reviewer #2: This paper addresses a timely and important issue, in the context of public health and nutrition transition in India, as well as equity in AI in healthcare. The study is strengthened by the large, representative dataset and the range of algorithms it utilized for analysis. Finally, its use of socioeconomic and demographic variables provide a nuanced context for the population being studied. There are a few key points that should be addressed and refined by the authors.

Can the authors define the acronym "NML" in the introduction?

Sorry, this is a typo. We meant "BMI" in the manuscript. Thank you for pointing this out.

Only adults aged 45 and above are included in the study. In the introduction, can the authors give more information on the dual public health problem of underweight/overweight in India? What are characteristics of this issue in the age group being studied compared to younger population subgroups? Why is this a priority age demographic?

Thank you for your suggestion. We agree that framing is important. We used this age bracket due to the limitation of LASI's dataset design—a nationally representative study of adults aged 45+ and their spouses. Moreover, in the Introduction section, we have now extended the background on India's "double burden" of malnutrition (coexisting under- and over-nutrition), noting that this burden is salient in later life due to the accumulation of chronic disease risks and functional limitations; we cite national evidence on rising overweight/obesity alongside persistent undernutrition to motivate the age focus.

Can the authors describe why they used SHAP values versus permutation importance when analyzing feature importance?

Thank you for raising this. We have added a methodological note clarifying why we prioritized SHAP for feature attribution. We prioritized SHAP because it provides locally consistent, additive attributions with a unique solution under Shapley axioms, enabling stable local-to-global interpretability for non-linear models such as gradient boosting and LightGBM. In contrast, permutation importance is model-agnostic but can be misleading under correlated predictors, often undervaluing features

that share information. Accordingly, we report SHAP as the primary attribution method and consider permutation-based results as complementary.

Did the authors analyze model performance by age subgroup? With an age range as substantial as this study's (45-116 years), I imagine there are distinct physiological differences between adults at various life stages. The paper would at minimum be strengthened by a discussion of this issue.

We appreciate this thoughtful observation. In our analytic sample, the vast majority of participants are between 45 and 75 years of age, approximately 90% (please see Supplementary Table 1). Our primary equity analyses emphasized caste and household economic status (MPCE) because these axes are structurally salient and policy-relevant in India and are central to the design and use of LASI and related ageing-health programs. However, this is an insightful point to be considered and we are glad to add this as a limitation to the Discussion section in our manuscript.

Can the authors include a brief 1-2 paragraph summary of their study population's demographic characteristics at the start of the results section? It would be useful for framing the findings from the machine learning analysis.

Thank you for the suggestion. We have now expanded the Descriptive Summary to elaborate on this.

Can the authors describe their rationale for choosing the selected bias mitigation methods?

Thank you for the question. We employed a portfolio of bias-mitigation strategies—Disparate Impact Remover, Reweighing, Exponentiated Gradient Reduction, Calibrated Equalized Odds post-processing, Reject Option Classification, and Equalized Odds post-processing—and evaluated them systematically rather than relying on a single method. We selected these techniques because they are widely used in the literature and are readily implemented in IBM's open-sourced AI Fairness 360 (AIF360) toolkit, facilitating transparent and reproducible workflows. We have added this rationale and supporting references to the manuscript.

The authors do not discuss the age of the participants as a limitation to the study's generalizability. This is challenging as India has a rapidly growing population that is demographically young. The author's thoughts on this study's relevance to younger populations and the limits of this study should be discussed at a minimum.

Thank you for your insight. This is indeed a crucial point that should be discussed: we have added this as limitations in our manuscript.
